# Investigation of AgInS_2_/ZnS Quantum Dots by Magnetic Circular Dichroism Spectroscopy

**DOI:** 10.3390/ma12213616

**Published:** 2019-11-04

**Authors:** Yulia Gromova, Anastasiia Sokolova, Danil Kurshanov, Ivan Korsakov, Victoria Osipova, Sergei Cherevkov, Aliaksei Dubavik, Vladimir Maslov, Tatiana Perova, Yurii Gun’ko, Alexander Baranov, Anatoly Fedorov

**Affiliations:** 1 School of Chemistry, Trinity College, University of Dublin, Dublin 2, Dublin, Ireland; 2 Center of informational optical technologies ITMO University, St. Petersburg 197101, Russia

**Keywords:** ternary quantum dots, magnetic circular dichroism, AgInS_2_/ZnS, AIS, electronic transitions

## Abstract

Over recent years, quantum dots (QDs) based on ternary metal dichalcogenides have attracted a lot of attention due to their unique properties and a range of potential applications. Here, we review the latest studies on the optical properties of AgInS_2_/ZnS QDs with emphasis on their theoretical modeling, and present our investigations of electronic transitions invisible in unstructured absorption spectra of AgInS_2_/ZnS QDs. The analysis of the absorption, photoluminescence excitation (PLE), and magnetic circular dichroism (MCD) spectra of hydrophobic and hydrophilic AgInS_2_/ZnS QDs of different sizes enables us to determine positions of electron transitions in these QDs. We demonstrate that the use of the second derivative of PLE spectra provides more unequivocal data on the position of the energy transitions compared with the second derivative of absorption spectra. Analysis of the MCD spectra reveals that the magnetic field induces energy level mixing in AgInS_2_/ZnS QDs in contrast to the traditional Cd-based QDs, where MCD is associated only with removing degeneracy of the excited energy level.

## 1. Introduction

Quantum dots (QDs) of ternary metal dichalcogenides (CuInS_2_ and AgInS_2_) are considered promising alternatives to binary cadmium and lead-containing chalcogenide QDs. Due to the low toxicity of ternary QDs, they are considered appropriate materials for the development of eco-friendly and bio-compatible devices [1,2,3,4,5,6,7]. The main feature of ternary QDs is their high tolerance to defect states, which leads to the formation of broadband absorption and emission. Other characteristic features of ternary QDs are giant Stokes shifts and long photoluminescence (PL) lifetimes of hundreds of nanoseconds (ns) [8,9]. It is known that CuInS_2_ and AgInS_2_ QDs possess similar properties. However, the Ag 4d^10^ orbital is lower in energy than the Cu 3d^10^ orbital and, as a result, Ag^I^ atoms can have both 6- and 4-coordination, while Cu^I^ atoms energetically favour 4-fold coordination [10]. This makes the structure of AgInS_2_ QDs more tolerant to the formation of defect states and provides additional opportunities for the tuning of their optical properties [11].

In this work, we concentrate on the investigation of an electronic structure of AgInS_2_ (AIS) QDs. We briefly summarize the current state of the art in the investigation of optical properties of AIS QDs and explain the basics of magnetic circular dichroism (MCD) as a tool for the investigation of electronic transitions in QDs. Then, in the research section, we present room temperature MCD spectra of AIS/ZnS QDs and analyse these data together with the second derivatives of absorption and photoluminescence excitation (PLE) spectra to provide more unequivocal information on the position of the energy transitions in AIS/ZnS QDs.

### 1.1. Optical Properties of AgInS_2_ Quantum Dots

There are several techniques used for the colloidal synthesis of AIS QDs, such as hot injection, chemical vapor deposition, thermal decomposition, etc. [12,13,14]. During colloidal synthesis, the size and optical properties of nanoparticles are adjusted by selecting the temperature and time of the reaction. A comprehensive review on the fabrication of AIS QDs has been previously published [15,16]. One of the challenges in ternary nanoparticle synthesis is the control of defect types and defect concentration. Even within a one-pot synthesized batch, individual QDs can have different defect distributions, leading to variation of QD optical properties, even in an ensemble with narrow size distribution [17].

**Figure 1:**
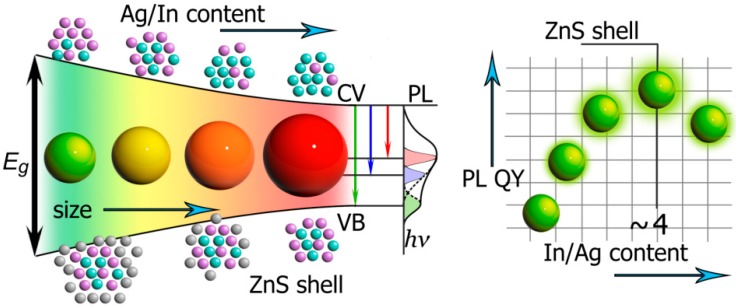
Representation of the tuning AIS QDs bandgap and PL quantum yield. Increasing of QD size and Ag/In content decrease bandgap energy (*E_g_*) and induce red shift in QD color. ZnS shell (as well as Zn-doping) works in the opposite way. The formation of broad PL spectra due to the presence of interband defect states is depicted in the middle of the sketch. Increasing In/Ag content, as well as ZnS shell growth, increase PL quantum yield (QY).

AIS QDs’ optical properties depend on QD size due to the quantum confinement effect [18,19]. However, the main tool for AIS QDs’ optical properties tuning is their composition change. For example, decreasing Ag/In ratio results in an increase in bandgap of the material and shifts absorption and PL bands to higher energies [1,20,21,22]. Another way to change the spectral properties of AIS QDs is by growing a semiconductor shell (ZnS usually [23]) or introducing Zn or Cu atoms to the QD core during the synthesis [24,25]. During the shell growth, core/shell materials are alloyed on the border and Zn atoms penetrate the QD cores. Since ZnS has a wider bandgap than AIS, alloying leads to increasing of the bandgap in QDs, and blue-shift of absorbance and PL spectra is observed. Also, the shell eliminates surface defects and as a result PL quantum yield increases [26]. In addition, the Ag/In ratio affects the PL quantum yield of QDs. The maximum value of PL quantum yield is achieved when Ag/In ratio is about ¼ [27,28]. This phenomenon was explained by the self-ordering of In sub-lattice in In-rich QDs [29]. In other words, the increase of PL quantum yield can be attributed to ordering of the structure of ternary QDs and reduction of intrinsic defects. This assumption correlates with other reports which state that the increasing In content also decreases QD PL lifetimes. It was observed that prolonged lifetimes in AIS QDs are associated with defects and defect elimination should decrease PL lifetime [1,27,28,29,30]. Figure 1 summarizes the options of optical properties tuning for AIS QDs: the influence of size and composition on QD size, PL colour, and PL quantum yield. The formation of broad PL spectra due to the presence of interband defect states is depicted in the middle of Figure 1. More details on manipulation by optical properties of AIS QDs can be found in the recent review [31].

### 1.2. Theoretical Models

Since the early days of research in this field, the donor-acceptor pair mechanism has been employed to explain the radiative recombination in both AgInS_2_ and CuInS_2_ nanocrystals [32,33,34]. The main idea of this mechanism is the radiative recombination of electrons and holes trapped by defect sites. Sulfur vacancies and interstitial silver atoms catch electrons and work as donors (*D*). Silver vacancies and interstitial sulfur atoms trap holes and operate as acceptors (*A*) [20,35]. This model was applied by Hamanaka et al. [34] to interpret the PL spectra of AIS QDs. For all defect-based models of PL, the Stokes shift depends on binding energies, which are related to donor and/or acceptor trapping.

Other theoretical models explain the optical properties of ternary QDs without evoking defect states. The mechanism of exciton self-trapping was proposed for AIS QDs [9,17,36]. Supposed electron-phonon interactions lead to a broadband emission, even in the case of a single nanocrystal. In this model, the magnitude of Stokes shift increases due to a large number of emitted phonons.

Shabaev et al. [37] developed a theoretical description for the structure of band-edge levels in spherical chalcopyrite CuInS_2_ nanocrystals and explain the Stokes shift up to 300 meV. In this approach, the emission is generated by the formally forbidden transition and has a long photoluminescence lifetime.

Recent theoretical models bring together several different mechanisms that previously have only been considered separately. The theoretical consideration of AIS QDs PL broadening presented in Ref [38] combines the features of several models:

- The possibility of the presence of both *D* and *A* sites in AIS QD [34]
- The strong electron–phonon interaction [36]
- The non-localized character of the electrons in the conduction band [17]

The theoretical model proposed in Ref. [38] is supported by spectroscopic studies. The authors demonstrate that PL broadening in AIS QD is associated with radiative transitions from energy states caused by defects localized at different positions within the QD volume. 

Therefore, three reasons for inhomogeneous broadening of AIS QD spectra could be named:

- Size polydispersity
- Dot-to-dot differences in chemical composition
- Different defect distribution within QDs

Another combined theoretical model explains the giant Stokes shift in AIS QDs [8]. The authors take into account electron-electron and electron-phonon interactions and use the density functional theory and method of invariants. The presence of at least one point defect responsible for hole trapping and formation of the localized polaron state was also considered. Numerical simulations show that the recombination of a non-trapped electron and a trapped hole gives a PL with the giant Stokes shift (~1 eV for AIS QDs with sizes of 1–2 nm); this is in good agreement with available experimental data [8,9,18,37].

### 1.3. Determination of the Bandgap in Ternary Quantum Dots

Typically, the AIS QDs demonstrate unstructured (i.e. without distinguishable bands) absorption spectra with the absorbance growing with increasing photon energy. However, increase of the In/Ag ratio over 4 leads to the formation of exciton-like features in the absorption spectra [11,27]. Absorption spectra of ternary materials reveal a long exponential tailing absorption below the band gap energy (*E_g_*), the so-called Urbach tail [39]. Urbach tail is almost a universal property of disordered solids, and is observed for alkali halides, II-IV compounds, III-V semiconductors, organics crystals, and amorphous systems. Urbach tail in ternary semiconductors is associated with structural defects [40].

Band gap energy of ternary QDs can be roughly estimated as the minima of a second derivative of the absorption spectra [41]. Otherwise $E_{g}$ can be estimated by replotting the spectra in Tauc coordinates for direct transitions ((*αhν*)^2^ vs *hν,* where *α* is the absorption coefficient constant and *hν* is the photon energy) and deducing the intercept of the spectra linear part with the abscissa [9,18,20,35]. However, since the typical absorption spectra of ternary QDs are very smooth, it is difficult to determine the band gap with the required accuracy based only on the analysis of the absorption spectra. Several technics for the determination of band gap energy in ternary QDs have recently been proposed, including methods based on the analysis of the PL spectra of QDs [42]. Band gap energy of AIS QDs was estimated from the PL spectral position and FWHM of PL band dependence on excitation energy [38]. At the same time, methods for the experimental determination of higher energy transitions in AIS QDs have not been reported. Attempts of investigation of high energy electron transition in AIS and AIS/ZnS QDs have been reported [43]. Researchers used the first derivative of absorption spectra for this purpose and compared their results with the modeled absorption of bulk AIS. However, the poor resolution of bands in absorbance spectra made this technique quite unreliable.

One of the ways to overcome poor resolutions of absorbance spectra is to use PLE spectra instead. PLE spectra allow to reduce inhomogeneous broadening due to the registration of a signal from a fraction of QDs with a small size variation. As a result, bands in PLE spectra are narrower and more distinguishable, when compared with the absorption spectra.

Recent studies underline the potential of circular dichroism (CD) [44,45] and magnetic circular dichroism (MCD) [46,47] spectroscopy for the investigation of an electronic energy structure of nananoparticles. MCD is a more universal technique, since it is applicable for nonchiral objects in contrast to CD. Basics of the MCD technique are discussed in detail below:

### 1.4. Magnetic Circular Dichroism Spectroscopy

MCD is the differential absorption of left and right circularly polarized light (lcp and rcp), measured in a sample placed in a magnetic field oriented parallel to the direction of the light propagation. MCD spectra contain information on the degeneracy and symmetry of the electronic levels, and paramagnetic properties of the studied systems [48,49].

**Figure 2:**
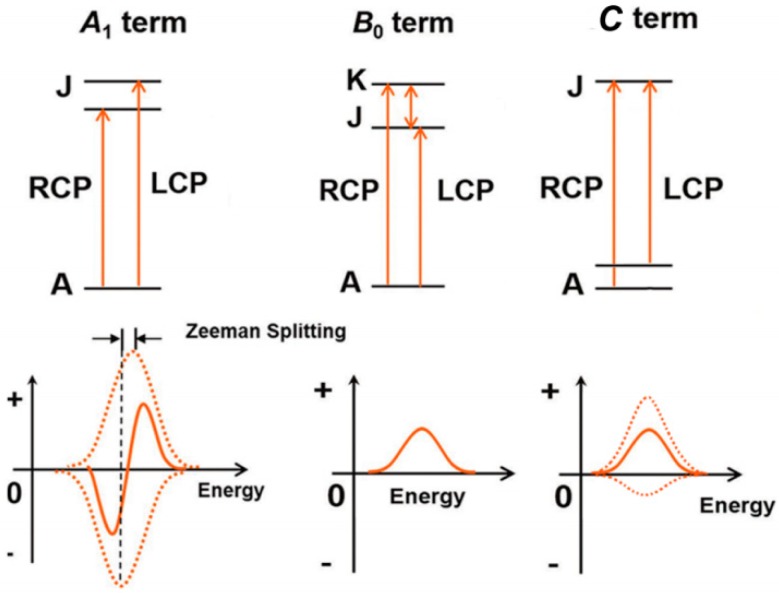
Scheme of band formation in magnetic circular dichroism spectra.

The bands in MCD spectra appear due to several reasons: (i) Zeeman splitting of degenerate excited states (so called A-term); (ii) magnetic ﬁeld induced mixing of the zero-ﬁeld states (B-term); (iii) distribution of Boltzmann population across a degenerate ground state (C-term). The scheme of MCD band formation is shown in Figure 2.

A-term has a highly distinctive first derivative band shape due to the energy shift between absorption bands of l cp and rcp light. In the case of A-term without admixture of other transitions, the zero-field transition position could be determined very precisely as an abscissa crossing point of MCD spectra or its second derivative. In samples without three-fold or higher rotation axis, the orbital degenerate states are absent and there is no Zeeman eﬀect (and A-terms) and therefore B-terms dominate the MCD spectrum. B-terms are also present in the spectra of high symmetry complexes, but tend to be signiﬁcantly less intense than the A and C terms. C term exhibits strong 1/*kT* temperature dependence and is usually associated with the presence of an open shell transition metal. When the splitting of the states is relatively small in the spectral bandwidths of the lcp and rcp light-specific absorption bands, the B and C terms have a Gaussian shape. A and B terms are normally investigated at ambient temperatures under moderate magnetic fields around 1-2 T, while superconducting magnets with ﬁeld strengths of 5–7 T and helium temperatures are often used to study terms.

MCD spectra for CdSe QDs were investigated for the first time in 1998 [50]. Recently MCD spectroscopy was successfully applied to study energy transitions in 2D CdSe nanoplatelets [46].

The MCD spectra of ternary QDs were reported only for CuIn-S_2_ QDs [51]. The MCD spectra were recorded for a temperature range of 3 to 50 K and magnetic field of 6 T. The MCD spectra of CuInS_2_ QDs represent a continuum of transitions with intensity growing at high energies. The only distinct feature was the temperature-dependent band (could be attributed to C-term). Authors associated this behavior with the presence of paramagnetic moments in nanocrystals that are coupled via *sp−d* exchange to the conduction and valence bands of the host CuInS_2_ lattice.

In this paper, we report, for the first time, the room temperature MCD spectra of AIS/ZnS QDs.

## 2. Materials and Methods 

### 2.1. Chemicals

Indium(III) chloride (InCl_3_, 99.9%), indium (III) acetate (In(OAc)_3_, 99.99%), silver nitrate (AgNO_3_, 99%), zinc(II) acetate dihydrate (Zn(OAc)_2_*2H_2_O, 99%), zinc stearate (technical grade), sodium sulfide (Na_2_S × 9H_2_O, 98%), NH_4_OH (aqueous 5.0 M solution), thioglycolic acid (TGA), 1-dodecanethiol (DDT, 98%), 1-octadecene (ODE, 90%), oleic acid (OA, 99%), acetone (>99%), and methanol (99.93%) were purchased from Sigma Aldrich and used without purification. All the aqueous solutions were prepared using Milli-Q water (Millipore) as a solvent.

### 2.2. Synthesis of Hydrophilic AgInS_2_/ZnS QDs

For a typical synthetic reaction, 1 mL of 0.1 M aqueous AgNO_3_ solution, 2 mL of 1.0 M aqueous TGA, and 0.2 mL of 5.0 M aqueous NH_4_OH solution were added to 92 mL of water under magnetic stirring and atmospheric pressure. The resulting turbid yellowish suspension changed after adding 0.45 mL of 5.0 M NH_4_OH solution and discolored after adding 0.9 ml of aqueous 1.0 M InCl_3_ solution containing 0.2 M HNO_3_. Then 1 mL of aqueous 1.0 M Na_2_S solution was added, and the solution was heated on a water bath at 90–95 °C for 30 min. Afterwards, 1 mL of 1.0 M aqueous TGA solution and 1 mL of aqueous 1.0 M Zn(OAc)_2_ solution containing 0.01 M HNO_3_ were added to the colloidal QD core solution to produce a ZnS shell. 

After the synthesis, the solution was cooled down to room temperature and concentrated using a rotary evaporation. For size-selection, 0.5 mL of isopropyl alcohol was added to initiate aggregation of the QDs with subsequent centrifugation at 10,000 rpm for 5 min. The precipitate was separated and designated further as fraction #1. This procedure was repeated until the solution was completely discolored. At each separation step, only the heaviest QD (with the biggest size) precipitate provided samples with the same composition, but different size. For our research, we have chosen the 6th and 8th fractions of QDs. For simplicity, we will refer to both samples as fraction 1 and 2.

### 2.3. Synthesis of Hydrophobic AgInS_2_/ZnS QDs

For a typical synthetic reaction, 0.018 g of AgNO_3_, 0.06 g of In(OAc)_3_, 8 mL 1-dodecantiol, and 210 µL were added in a three-necked round bottom flask equipped with a magnetic stirring bar and heated under vacuum at 135 °C for 30 min. Then, mixture was heated up to 185 °C in argon atmosphere, and the mixture was kept at this temperature for 15 min until a bright red solution was formed.

The core AgInS_2_ QDs were covered with a ZnS shell. To achieve this, the mixture of 0.02 g of zinc stearate and of 4 mL of octadecene was degassed under vacuum for 30 min at 120 °C under magnetic stirring. The solution was then heated to 160 °C in an argon atmosphere until complete zinc stearate dissolution. For shell growth, 2.5 mL of Zn solution was injected into the core, and then 0.5 mL of zinc precursor was injected every 15 minutes. The solution was heated to 210 °C by magnetic stirring for 45 min.

After the synthesis, the solution was cooled down to room temperature and 3 mL of acetone was added to initiate aggregation of the QDs with subsequent centrifugation at 10,000 rpm for 5 min. The precipitate was separated and designated further as fraction #1. This procedure was repeated 4 times until the solution was completely discolored. We used the 2nd and 4th fractions for our research. For simplicity, we will refer to both samples as fraction 1 and 2.

### 2.4. Methods

Absorption spectra were measured with a Shimadzu UV-3600 spectrophotometer. Photoluminescence spectra were registered using Cary Eclipse (Agilent) fluorescence spectrofluorimeter and photoluminescence excitation spectra were recorded at the maxima of PL bands with spectrofluorimeter Fluorat-02-Panorama. Jasco J-1500 CD spectrophotometer and electromagnet MCD-581 with field of up to 1.5 Tesla were used for MCD spectra measurements.

### 2.5. Data Treatment

To reduce machine artifacts in MCD spectra that originated from baseline imperfections, we treated MCD spectra as follows: (i) calculated a correction spectrum as a half of the sum of MCD spectra recorded under magnetic field of an opposite sign and (ii) subtracted the obtained correction spectrum from the original MCD spectra. This procedure is relevant, since non-chiral samples demonstrate a mirror image MCD spectra in the opposite magnetic field. 

Before the calculation of the second derivative, PLE, absorbance and MCD spectra were smoothed with the Savitsky-Golay method with points of window parameter between 25 to 55 for different spectra. After the calculation, the second derivatives were smoothed with points of window parameter between 50 to 125. The numbers of points of window were chosen manually for each spectrum to ensure better noise reduction, together with preservation of spectral details.

## 3. Results and Discussion

**Figure 3:**
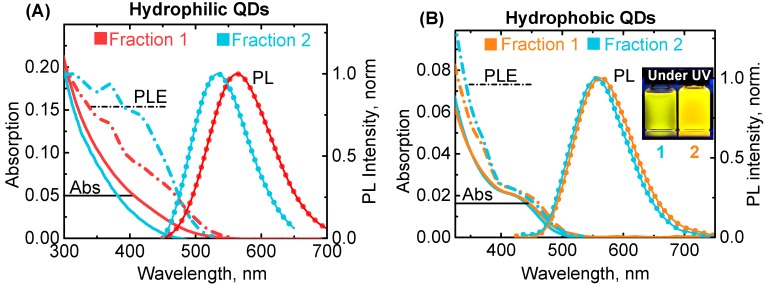
Photoluminescence and photoluminescence excitation spectra for two fractions of hydrophilic (**A**) and hydrophobic (**B**) AIS/ZnS QDs. PL excitation was done at 405 nm. PLE was registered at the maximum of PL (562 nm and 532 nm for Fraction 1 and Fraction 2 of hydrophilic QDs, respectively, and 560 and 550 nm for Fraction 1 and Fraction 2 of hydrophobic QDs, respectively). The images of hydrophobic QD solutions taken under a UV lamp are presented in the insert.

We synthesized AIS/ZnS QDs by two different approaches in organic solvents [13] and in an aqueous solution [18] to ensure the universality of our results. The selected synthesis methods allowed the producing of QD ensembles with broad size distribution. The selective size separation of QD ensembles produced a number of QD fractions with slightly different mean sizes. For the present investigation, we chose two fractions with the most distinguishable bands in MCD spectra for each hydrophobic and hydrophilic QD. Such an approach, instead of varying QDs size by temperature change, enabled to ensure the identity of the Ag/In ratio within each batch and guarantee that all spectral changes are associated only with differences in size, not composition. Figure 3 shows photoluminescence (PL), absorption, and PL excitation (PLE) spectra of the studied samples. Both hydrophilic and hydrophobic QDs have broad PL bands with full width at half maximum (FWHM) of about 420–430 meV. The distance between PL band maxima of the investigated fractions of hydrophilic and hydrophobic QDs are 140 meV and 65 meV, respectively. Despite the small shift between PL bands of hydrophobic QDs, the difference in PL colour of these two fractions is clearly visible to the naked eye (the photo of the samples under UV lamp is presented in an insert in Figure 3). According to the literature, AgInS_2_/ZnS QDs with such composition and spectral properties have sizes between 3 nm and 4 nm [13,18]. Absorption spectra of hydrophilic QDs are featureless, while hydrophobic QDs possess a pronounced band similar to the fundamental excitonic transition in CdSe QDs. According to the literature, a noticeable excitonic band appears in AIS QDs when In/Ag ratio is over ~4 [11,27]. The ratio of In/Ag precursors was 2.8 and 3.7 for hydrophobic and hydrophilic QDs, respectively; therefore, the change in absorbance spectra could be associated with the difference in indium content. 

PLE spectra registered at the maximum of PL bands reveal some additional transitions unresolved in the absorption spectra.

**Figure 4:**
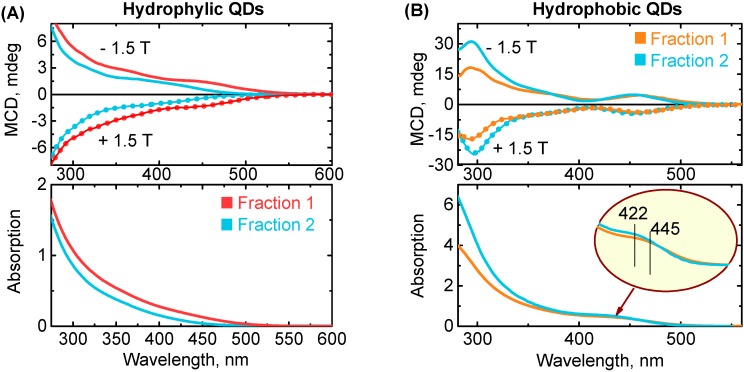
**Magnetic circular dichroism** (top) and corresponding absorption (bottom) spectra of two fractions of hydrophilic (**A**) and hydrophobic (**B**) AIS/ZnS QDs.

To obtain additional information on electronic transitions in QDs, we applied MCD spectroscopy. MCD spectra were recorded under a magnetic field of +1.5 and −1.5 Tesla aligned with the direction of light propagation. MCD spectra recorded under opposite magnetic field represent a mirror image of each other and are very similar to MCD spectra reported for CIS QDs [51]. MCD spectra present the rising (or decreasing, depends on field sign) continuum of transitions with a number of broad bands invisible in the absorption spectra (Figure 4).

**Figure 5:**
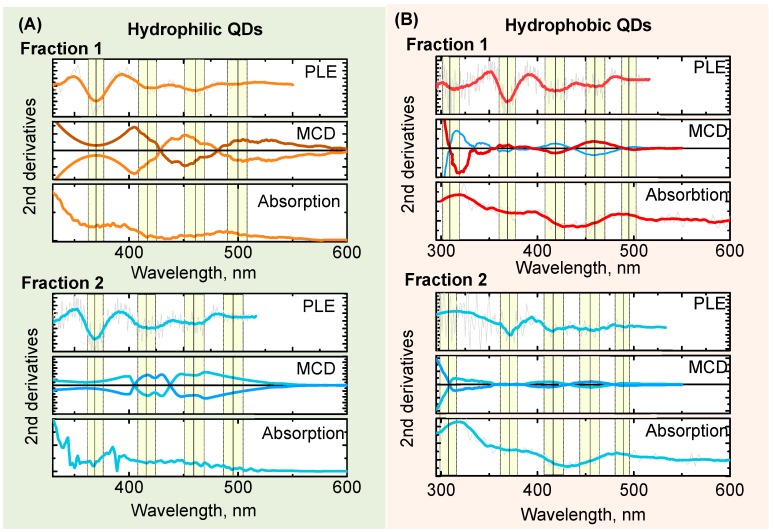
The second derivatives of PLE (top), magnetic circular dichroism (middle), and absorption (bottom) spectra for each fraction of hydrophilic (**A**) and hydrophobic (**B**) AgInS_2_/ZnS QDs. The position of electronic transitions is determined as positions of second derivatives minimums, marked by vertical lines with error within yellow bars.

To determine the position of the electronic transition in QD spectra, we analyzed the second derivatives of the absorption and PLE spectra (Figure 5). In the absorption and PLE spectra, all bands could be approximated by Gaussians with maxima at transition positions. Therefore, the local minima of the second derivatives of absorption and PLE spectra reveal the position of transitions.

Analysis of spectra began in the bandgap area. According to the literature [43,44], the position of the fundamental excitonic transition corresponds to the first minimum of the 2nd derivative of absorbance spectra. Comparison of the 2nd derivatives of absorbance and PLE spectra reveals that a broad minimum in absorbance corresponds to two minima in PLE (at 456–461 nm and at 412–416 nm, respectively). The positions of both transitions are size-dependent: both are blue-shifted for the smaller fraction of QDs, which allows to attribute both transition to excitonic energy levels.

Interestingly, according to PLE spectra, there is another size-dependent transition below the bandgap. Such transition was also observed in CuInS_2_ QDs [41]. Existence of this transition was explained by inversion of the 1S and 1P hole levels at the top of the valence band [37]. However, theoretical calculations made for AIS QDs do not demonstrate this inversion and postulate that 1S(h)–1S(e) is an optical active excitonic transition with the lowest energy [8]. The Stock shift between the first observed transition and maximum of PL band is only 0.2–0.3 eV, which is much smaller than the typical Stock shifts reported for AIS/ZnS QDs of 0.4–0.8 eV [18,43,44,52]. Therefore, the observed transition might be attributed to absorption of defect states within the bandgap.

Forward transition at the 369 nm (3.36 eV) is positioned independent of the QD size and Ag/In ratio. This makes its origin questionable for now.

Another size-independent transition is observed in hydrophobic QDs at 309 nm (4.01 eV). This could be related to ZnS shell [43]. The absence of this peak for hydrophilic QDs could be associated with different shell thickness.

All transition positions are given in Table 1.

Analysis of MCD spectra is complicated since A- and B- terms are strongly overlapped. According to [37], the transitions in ternary QDs are degenerate, offering a distinctive A-term in MCD spectra. Furthermore, previous results [46,50] show that the two first transitions in CdSe QDs are associated with A-terms only. However, symmetry breaking in AIS QDs due to shape imperfections and the presence of defect sites could lead to the appearance of B-terms. In this situation, B-terms associated with the mix of energy levels under a magnetic field should dominate. Another reason for B-term appearance could be the high density of nondegenerate energy states from defect sites.

## 4. Conclusions

Energy transitions in AIS/ZnS QDs have been investigated using new approaches. We demonstrated that by using PLE spectra, we can gain more detailed information on electronic transitions in AIS QDs, when compared with using absorbance spectra only. The results are in good agreement with the theoretical prediction of the excitonic fine structure developed for Cu-In-S_2_ QDs.

The analysis of MCD spectra revealed that a magnetic field induces in AIS QDs energy level mixing (B-term) in contrast to CdSe QDs, where the MCD of low energy levels is associated only with removing degeneracy of the excited energy level (A-term). 

We believe that this research will be useful for further understanding of the electronic structure and properties of quantum nanostructures, which are based on ternary metal dichalcogenides.

## Figures and Tables

**Table 1:** Transition positions in nm (eV), type of magnetic circular dichroism term.

No.	Hydrophilic			Hydrophobic		
	Fraction 1	Fraction 2	MCD Term	Fraction 1	Fraction 2	MCD Term
1	499 (2.48)	496 (2.49)	A + B	495 (2.50)	489 (2.53)	A + B
2	461 (2.69)	459 (2.70)	A + B	458 (2.70)	456 (2.72)	B
3	417 (2.97)	415 (2.98)	A + B	418 (2.96)	412 (3.01)	B
4	369 (3.36)	370 (3.35)	unknown	369 (3.36)	369 (3.36)	unknown
5	-	-	-	309 (4.01)	309 (4.01)	A

## References

[1] Regulacio M.D., Win K.Y., Lo S.L., Zhang S.-Y., Zhang X., Wang S., Han M.-Y., Zheng Y. (2013). Aqueous synthesis of highly luminescent AgInS2-ZnS quantum dots and their biological applications. Nanoscale.

[2] Mir I.A., Bhat M.A., Muhammad Z., Rehman S.U., Hafeez M., Khan Q., Zhu L. (2019). Differential and comparative sensing modes of AIS and AIS@ZnS core-shell quantum dots towards bioanalytes. J. Alloys Compd..

[3] Martynenko I.V., Kusic D., Weigert F., Stafford S., Donnelly F.C., Evstigneev R., Gromova Y., Baranov A.V., Rühle B., Kunte H.-J. (2019). Magneto-fluorescent microbeads for bacteria detection constructed from superparamagnetic Fe3O4 nanoparticles and AIS/ZnS quantum dots. Anal. Chem..

[4] Nong J., Lan G., Jin W., Luo P., Guo C., Tang X., Zang Z., Wei W. (2019). Eco-friendly and high-performance photoelectrochemical anode based on AgInS 2 quantum dots embedded in 3D graphene nanowalls. J. Mater. Chem. C.

[5] Li Y., Li Z., Ye W., Zhao S., Yang Q., Ma S., Xiao G., Liu G., Wang Y., Yue Z. (2019). Gold nanorods and graphene oxide enhanced BSA-AgInS 2 quantum dot-based photoelectrochemical sensors for detection of dopamine. Electrochim. Acta.

[6] Mansur A.A.P., Mansur H.S., Tabare C., Paiva A., Capanema N.S.V. (2019). Eco-friendly AgInS2/ZnS quantum dot nanohybrids with tunable luminescent properties modulated by pH-sensitive biopolymer for potential solar energy harvesting applications. J. Mater. Sci. Mater. Electron..

[7] Politano G.G., Cazzanelli E., Versace C., Vena C., De Santo M.P., Castriota M., Ciuchi F., Bartolino R. (2018). Graphene oxide on magnetron sputtered silver thin films for SERS and metamaterial applications. Appl. Surf. Sci..

[8] Baimuratov A.S., Martynenko I.V., Baranov A.V., Fedorov A.V., Rukhlenko I.D., Kruchinin S.Y. (2019). Giant Stokes Shifts in AgInS 2 Nanocrystals with Trapped Charge Carriers. J. Phys. Chem. C.

[9] Stroyuk O., Raevskaya A., Spranger F., Selyshchev O., Dzhagan V., Schulze S., Zahn D.R.T., Eychmuller A. (2018). Origin and Dynamics of Highly Efficient Broadband Photoluminescence of Aqueous Glutathione-Capped Size-Selected Ag-In-S Quantum Dots. J. Phys. Chem. C.

[10] Xiao Z., Du K.Z., Meng W., Mitzi D.B., Yan Y. (2017). Chemical Origin of the Stability Difference between Copper(I)- and Silver(I)-Based Halide Double Perovskites. Angew. Chemie Int. Ed..

[11] Hong S.P., Park H.K., Oh J.H., Yang H., Do Y.R. (2012). Comparisons of the structural and optical properties of o-AgInS2, t-AgInS2, and c-AgIn5S8 nanocrystals and their solid-solution nanocrystals with ZnS. J. Mater. Chem..

[12] Wang D., Zheng W., Hao C., Peng Q., Li Y. (2008). General synthesis of I-III-VI2 ternary semiconductor nanocrystals. Chem. Commun..

[13] Chen S., Ahmadiantehrani M., Zhao J., Zhu S., Mamalis A.G., Zhu X. (2016). Heat-up synthesis of Ag-In-S and Ag-In-S/ZnS nanocrystals: Effect of indium precursors on their optical properties. J. Alloys Compd..

[14] Saji P., Ganguli A.K., Bhat M.A., Ingole P.P. (2016). Probing the Crystal Structure, Composition-Dependent Absolute Energy Levels, and Electrocatalytic Properties of Silver Indium Sulfide Nanostructures. ChemPhysChem.

[15] Girma W.M., Fahmi M.Z., Permadi A., Abate M.A., Chang J.Y. (2017). Synthetic strategies and biomedical applications of I-III-VI ternary quantum dots. J. Mater. Chem. B.

[16] Moodelly D., Kowalik P., Bujak P., Pron A., Reiss P. (2019). Synthesis, photophysical properties and surface chemistry of chalcopyrite-type semiconductor nanocrystals. J. Mater. Chem. C.

[17] Zang H., Li H., Makarov N.S., Velizhanin K.A., Wu K., Park Y.-S., Klimov V.I. (2017). Thick-Shell CuInS2/ZnS Quantum Dots with Suppressed “Blinking” and Narrow Single-Particle Emission Line Widths. Nano Lett..

[18] Raevskaya A., Lesnyak V., Haubold D., Dzhagan V., Stroyuk O., Gaponik N., Zahn D.R.T., Eychmüller A. (2017). A Fine Size Selection of Brightly Luminescent Water-Soluble Ag-In-S and Ag-In-S/ZnS Quantum Dots. J. Phys. Chem. C.

[19] Kurshanov D.A., Gromova Y.A., Cherevkov S.A., Ushakova E.V., Kormilina T.K., Dubavik A., Fedorov A.V., Baranov A.V. (2018). Non-Toxic Ternary Quantum Dots AgInS 2 and AgInS 2 /ZnS: Synthesis and Optical Properties. Opt. Spectrosc..

[20] Dai M., Ogawa S., Kameyama T., Okazaki K.-I., Kudo A., Kuwabata S., Tsuboi Y., Torimoto T. (2012). Tunable photoluminescence from the visible to near-infrared wavelength region of non-stoichiometric AgInS 2 nanoparticles. J. Mater. Chem..

[21] Torimoto T., Kameyama T., Kuwabata S. (2014). Photofunctional materials fabricated with chalcopyrite-type semiconductor nanoparticles composed of AgInS2 and its solid solutions. J. Phys. Chem. Lett..

[22] Kobosko S.M., Kamat P.V. (2018). Indium-Rich AgInS2-ZnS Quantum Dots—Ag-/Zn-Dependent Photophysics and Photovoltaics. J. Phys. Chem. C.

[23] Chen T., Hu X., Xu Y., Wang L., Jiang W., Jiang W., Xie Z. (2019). Hydrothermal synthesis of highly fluorescent Ag–In–S/ZnS core/shell quantum dots for white light-emitting diodes. J. Alloys Compd..

[24] Zhang H., Fang W., Zhong Y., Zhao Q. (2019). Zn-Ag-In-S quantum dot sensitized solar cells with enhanced efficiency by tuning defects. J. Colloid Interface Sci..

[25] Raevskaya A., Rozovik O., Novikova A., Selyshchev O., Stroyuk O., Dzhagan V., Goryacheva I., Gaponik N., Zahn D.R.T., Eychmuller A. (2018). Luminescence and photoelectrochemical properties of size-selected aqueous copper-doped Ag-In-S quantum dots. RSC Adv..

[26] Liao S., Huang Y., Zhang Y., Shan X., Yan Z., Shen W. (2015). Highly enhanced photoluminescence of AgInS 2 /ZnS quantum dots by hot-injection method. Mater. Res. Express.

[27] Xiang W., Xie C., Wang J., Zhong J., Liang X., Yang H., Luo L., Chen Z. (2014). Studies on highly luminescent AgInS2 and Ag-Zn-In-S quantum dots. J. Alloys Compd..

[28] Chang J.Y., Wang G.Q., Cheng C.Y., Lin W.X., Hsu J.C. (2012). Strategies for photoluminescence enhancement of AgInS 2 quantum dots and their application as bioimaging probes. J. Mater. Chem..

[29] Marin G., Marquez R., Guevaraba R., Wasim S.M., Delgado J.M., Rincón C., Pérez G.S., Molina I., Bocaranda P. (2000). Crystal Growth, Structural and Optical Characterization of the Ordered Vacancy Compounds of the I-III 3 -VI 5 and I-III 5 -VI 8 Families. Jpn. J. Appl. Phys..

[30] Wang L., Kang X., Pan D. (2017). Gram-Scale Synthesis of Hydrophilic PEI-Coated AgInS2 Quantum Dots and Its Application in Hydrogen Peroxide/Glucose Detection and Cell Imaging. Inorg. Chem..

[31] Yarema O., Yarema M., Wood V. (2018). Tuning the Composition of Multicomponent Semiconductor Nanocrystals: The Case of I-III-VI Materials. Chem. Mater..

[32] Kolny-Olesiak J., Weller H. (2013). Synthesis and application of colloidal CuInS2 semiconductor nanocrystals. ACS Appl. Mater. Interfaces.

[33] Van Der Stam W., Berends A.C., De Mello Donega C. (2016). Prospects of Colloidal Copper Chalcogenide Nanocrystals. ChemPhysChem.

[34] Hamanaka Y., Ogawa T., Tsuzuki M., Kuzuya T. (2011). Photoluminescence properties and its origin of AgInS2 quantum dots with chalcopyrite structure. J. Phys. Chem. C.

[35] Tsuji I., Kato H., Kobayashi H., Kudo A. (2004). Photocatalytic H2 evolution reaction from aqueous solutions over band structure-controlled (Agln)xZn2(1-x)S2 solid solution photocatalysts with visible-light response and their surface nanostructures. J. Am. Chem. Soc..

[36] Stroyuk O., Weigert F., Raevskaya A., Spranger F., Würth C., Resch-Genger U., Gaponik N., Zahn D.R.T. (2019). Inherently Broadband Photoluminescence in Ag–In–S/ZnS Quantum Dots Observed in Ensemble and Single-Particle Studies. J. Phys. Chem. C.

[37] Shabaev A., Mehl M.J., Efros A.L. (2015). Energy band structure of CuInS2 and optical spectra of CuInS2 nanocrystals. Phys. Rev. B Condens. Matter Mater. Phys..

[38] Martynenko I.V., Baimuratov A.S., Weigert F., Soares J.X., Dhamo L., Nickl P., Doerfel I., Pauli J., Rukhlenko I.D., Baranov A.V. (2019). Photoluminescence of Ag-In-S/ZnS quantum dots: Excitation energy dependence and low-energy electronic structure. Nano Res..

[39] Urbach F. (1953). The long-wavelength edge of photographic sensitivity and of the electronic Absorption of Solids. Phys. Rev..

[40] Bonalde I., Medina E., Rodriguez M., Wasim S.M., Marin G., Rincon C., Rincón A., Torres C. (2004). Urbach tail, disorder, and localized modes in ternary semiconductors. Phys. Rev. B Condens. Matter Mater. Phys..

[41] Nagamine G., Nunciaroni H.B., McDaniel H., Efros A.L., Cruz C.H.D.B., Padilha L.A. (2018). Evidence of Band-Edge Hole Levels Inversion in Spherical CuInS2 Quantum Dots. Nano Lett..

[42] Fuhr A.S., Yun H.J., Makarov N.S., Li H., McDaniel H., Klimov V.I. (2017). Light Emission Mechanisms in CuInS2 Quantum Dots Evaluated by Spectral Electrochemistry. ACS Photonics.

[43] Cichy B., Rich R., Olejniczak A., Gryczynski Z., Strek W. (2016). Two blinking mechanisms in highly confined AgInS2 and AgInS2/ZnS quantum dots evaluated by single particle spectroscopy. Nanoscale.

[44] Martynenko I.V., Baimuratov A.S., Osipova V.A., Kuznetsova V.A., Purcell-Milton F., Rukhlenko I.D., Fedorov A.V., Resch-Genger U., Baranov A.V., Gun’Ko Y.K. (2018). Excitation Energy Dependence of the Photoluminescence Quantum Yield of Core/Shell CdSe/CdS Quantum Dots and Correlation with Circular Dichroism. Chem. Mater..

[45] Kuznetsova V., Visheratina A., Ryan A., Martynenko I., Loudon A., Maguire C.M., Orlova A., Baranov A., Fedorov A., Volkov Y. (2017). Enantioselective cytotoxicity of ZnS:Mn quantum dots in A549 cells. Chirality.

[46] Gromova Y.A., Miropoltsev M.A., Cherevkov S.A., Maslov V.G., Baranov A.V., Fedorov A.V. (2018). Magnetic Circular Dichroism in 2D Colloidal Semiconductor Nanocrystals. Opt. Spectrosc..

[47] Gromova Y.A., Maslov V.G., Baranov M.A., Serrano-Garcia R., Kuznetsova V.A., Purcell-Milton F., Baranov A.V., Fedorov A.V., Gun’Ko Y.K., Gun’Ko Y.K. (2018). Magnetic and Optical Properties of Isolated and Aggregated CoFe2O4 Superparamagnetic Nanoparticles Studied by MCD Spectroscopy. J. Phys. Chem. C.

[48] Stephens P.J. (1970). Theory of magnetic circular dichroism. J. Chem. Phys..

[49] Solomon E.I., Pavel E.G., Loeb K.E., Campochiaro C. (1995). Magnetic circular dichroism spectroscopy as a probe of the geometric and electronic structure of non-heme ferrous enzymes. Coord. Chem. Rev..

[50] Kuno M., Nirmal M., Bawendi M.G., Efros A., Rosen M. (1998). Magnetic circular dichroism study of CdSe quantum dots. J. Chem. Phys..

[51] Rice W.D., McDaniel H., Klimov V.I., Crooker S.A. (2014). Magneto-optical properties of CuInS2 nanocrystals. J. Phys. Chem. Lett..

[52] Gabka G., Bujak P., Giedyk K., Ostrowski A., Malinowska K., Herbich J., Golec B., Wielgus I., Pron A. (2014). A simple route to alloyed quaternary nanocrystals Ag-In-Zn-S with shape and size control. Inorg. Chem..

